# Polyphenols and Fish Oils for Improving Metabolic Health: A Revision of the Recent Evidence for Their Combined Nutraceutical Effects

**DOI:** 10.3390/molecules26092438

**Published:** 2021-04-22

**Authors:** Lucía Méndez, Isabel Medina

**Affiliations:** Instituto de Investigaciones Marinas-Consejo Superior de Investigaciones Científicas (IIM-CSIC), Eduardo Cabello 6, E-36208 Vigo, Spain; medina@iim.csic.es

**Keywords:** eicosapentaenoic acid, docosahexaenoic acid, omega-3 polyunsaturated fatty acids, plant bioactives, oxidative stress, inflammation, metabolic disorders, nutraceuticals

## Abstract

Polyphenols and omega-3 polyunsaturated fatty acids from fish oils, i.e., eicosapentaenoic and docosahexaenoic acids, are well-recognized nutraceuticals, and their single antioxidant and anti-inflammatory properties have been demonstrated in several studies found in the literature. It has been reported that the combination of these nutraceuticals can lead to three-fold increases in glutathione peroxidase activity, two-fold increases in plasma antioxidant capacity, decreases of 50–100% in lipid peroxidation, protein carbonylation, and urinary 8-isoprotanes, as well as 50–200% attenuation of common inflammation biomarkers, among other effects, as compared to their individual capacities. Therefore, the adequate combination of those bioactive food compounds and their single properties should offer a powerful tool for the design of successfully nutritional interventions for the prevention and palliation of a plethora of human metabolic diseases, frequently diet-induced, whose etiology and progression are characterized by redox homeostasis disturbances and a low-grade of chronic inflammation. However, the certain mechanisms behind their biological activities, in vivo interaction (both between them and other food compounds), and their optimal doses and consumption are not well-known yet. Therefore, we review here the recent evidence accumulated during the last decade about the cooperative action between polyphenols and fish oils against diet-related metabolic alterations, focusing on the mechanisms and pathways described and the effects reported. The final objective is to provide useful information for strategies for personalized nutrition based on these nutraceuticals.

## 1. Introduction

Nutraceuticals, defined as “any substance that is food or part of a food and provide medical or health benefits, including the prevention and treatment of disease” [1], are currently considered a viable strategy for the prevention and palliation of several human diseases, including those induced by the consumption of “obesogenic” diets.

The widespread consumption of diets high in saturated fat and sugar and low in polyunsaturated fatty acids (PUFAs) and antioxidants, together with a more sedentary lifestyle, have notably increased the prevalence of metabolic alterations such as the so-called Metabolic Syndrome (MetS) [2]. MetS is a pathological condition defined by the simultaneous presence of different combinations of three or more of the following metabolic alterations: abdominal obesity, blood hypertension, hyperglycemia, and serum dyslipidemia [3]. It has been estimated that MetS affects over a billion people worldwide [4] and is considered a risk factor for noncommunicable diseases such as type 2 diabetes and cardiovascular diseases (CVD) [5]. Other main chronic illnesses promoted by obesity are cancer and neurodegenerative pathologies, both affecting a great number of people worldwide and whose incidence grows in parallel with the dramatic increase in the aging population around the world [6].

All the metabolic alterations of MetS and its derived diseases are characterized by an increase in oxidative stress and a proinflammatory status [7]. The pathogenesis of MetS is very complex and not fully understood yet, but the breakdown of the redox homeostasis induced by the imbalance between prooxidants and antioxidants might play important roles in its development [8] and favor the appearance of inflammation and the other pathophysiological alterations concomitant with the syndrome [9]. Interestingly, it seems that oxidative stress and inflammation also are both the cause and the consequence of MetS and associated diseases [10].

For this reason, bioactive compounds naturally present in food with antioxidant and/or anti-inflammatory properties have attracted the attention of the scientific community for the development of successful nutritional interventions. Among those, dietary polyphenols are often considered because they are one of the most important groups of natural antioxidants and anti-inflammatory agents found in human diets, including fruits, vegetables, grains, tea, essential oils, and their derived foods and beverages [11]. More than 10,000 structural variants exist in the polyphenol family [12], more than 500 in foodstuffs [13]. According to their chemical structures, they can be classified into phenolic acids, flavonoids, stiblins, phenolic alcohols, and lignans [14]. This heterogeneity determines diverse bioavailability, biological functions, and health effects and should be considered in studies concerning polyphenols [15].

As potent antioxidants and antimicrobial agents, polyphenols have been used as food additives because they are natural food ingredients and hence, safe and available [16]. Interestingly, different polyphenols of diverse origins (tea, tomato, pomegranate, pistachio, or grape) have been often used as additives in fish and fish product foodstuffs. In those cases, polyphenols have demonstrated that their antioxidant properties were able to prevent lipid oxidation of PUFAs, avoiding organoleptic and sensory deterioration, formation of toxic substances, and above all, preserve the bioactive and functional properties of omega-3 PUFAs of marine origin [17,18]. Moreover, the combination between polyphenols and omega-3 PUFAs reciprocally influence their pharmacological profiles and bioavailability because polyphenols prevent omega-3 PUFAs oxidation, assisting their intestinal uptake while preserving their bioactivity [19,20], and omega-3 PUFAs influence the metabolism and bioaccesibility of polyphenols [21,22] and when they are conjugated (as lipophenols) they can have, among others, the following potential advantages: increase lipophilicity, cell penetration, and bioavailability of specific polar phenolic drugs, reach appropriate solubility of hydrophobic drugs and can become tissue/tumor-specific, limit auto-oxidation of the conjugate PUFAs and mask their hydroxyl polar functions and thus, by reducing their biotransformation or the pace of oxidative degradation, increase antioxidant properties, etc. [23].

Omega-3 PUFAs, i.e., eicosapentaenoic (EPA) and docosahexaenoic (DHA) acids are essential dietary PUFAs with numerous health benefits. Humans and other mammalian can synthesize EPA and DHA via the activity of elongase and desaturase enzymes over the shorter-chain omega-3 fatty acids, α-linolenic acid (ALA), which is usually found in many land plants commonly part of the normal human diet. However, it has been described that this de novo synthesis process is poorly efficient, making it necessary to obtain adequate amounts of EPA and DHA from the diet, mainly through the consumption of fish and seafood products [24].

Habitual intake of fish-derived omega-3 has been reported to be involved in the prevention of several metabolic alterations and diseases, including autoimmune disorders, MetS, diabetes, CVD, neurodegenerative diseases, cancer, and others [25]. Those beneficial effects are the consequence of their biological functions and bioactivities. EPA and DHA are incorporated in many parts of the body, including cell membranes, altering their fluidity and viscosity [26]. They also interact with several transcription factors like peroxisome proliferator-activated receptor (PPAR), nuclear factor kB (NF-kB), or sterol regulatory element-binding proteins (SREBP), affecting critical metabolic pathways [27]. Interestingly, EPA and DHA play an important role in anti-inflammatory processes because they are substrates for cyclooxygenase, lipoxygenase, cytochrome P450, and several other enzymes [28]. As a result, there are generated numerous lipid mediators involved in inflammatory processes, including specialized pro-resolving lipid mediators (SPMs), which actively resolve inflammation [29].

Several studies have pointed out that fish oils can also improve the antioxidant defense, mainly via nuclear factor erythroid 2 (Nrf2)-dependent mechanisms [30,31,32,33]. However, the influence of highly unsaturated fatty acids on redox homeostasis remains controversial. The high consumption of these omega-3 causes an enriched in PUFAs of cell membranes and tissues, which could make them more vulnerable to suffering from lipid peroxidation under oxidative stress insults because of the presence of those high unsaturated structures carrying many “fragile” double bonds [34].

The combination of omega-3 PUFAs and polyphenols in food may not only be useful for retaining their bioactive value and bioavailability but also may eliminate, or at least limiting, the deleterious potential effects of the high PUFAs intake regarding oxidation. Moreover, a recent genome-wide association study (GWAS) [35] showed that both polyphenols (particularly resveratrol and flavonoids) and omega-3 PUFAs can potentially regulate genes implicated in immune responses and disease pathways, but also several individual effects on gene expression have been reported. As a consequence, the coadministration of these nutraceuticals may result in additive (A + B = C; A = 1 and B = 1; C = 2), synergistic (A + B = C; A = 0 and B = 0; C > 0), amplificated (A + B = C; A = 1 and B = 1; C > 2), potentiated (A + B = C; A = 0 and B = 1; C > 1), or even attenuated (A + B = C; A = 1 and B = 1; C < 2), and antagonist (A + B = C; A = 1 and B = 1; C = 0) health effects that need to be clarified [36,37,38]. It is necessary to unequivocally identify the molecular processes and biochemical pathways that can be modulated by the combination of those nutrients, determine the optimal consumption in the context of several metabolic alterations and describe potential adverse or antagonist effects that could exist, among other concerns.

Therefore, the aim of this review was to summarize the recent evidence that supports the combined use of fish-derived PUFAs and polyphenols as nutraceuticals for the prevention and treatment of metabolic disturbances characterized by oxidative stress and inflammation. For this reason, we have searched the studies published during the last decade (since 2010) that have investigated the effects that the dietary coadministration of polyphenols and fish oil or fish-derived omega-3 exerts on health. We have only considered the studies that tested the health action of the omega-3 PUFAs for marine origin, EPA, and DHA, excluding those that only evaluated the effects of their precursor ALA, and/or other PUFAs different from fish-derived omega-3. Moreover, those studies addressing the role of the combination between polyphenols and fish-derived omega-3 PUFAs as food additives were also omitted from the review.

## 2. Combined Polyphenols and Fish Oils Intake for Improving Metabolic Health

During the last decade, several researchers have investigated the synergistic, additive, and complementary effects between fish oils (EPA and DHA) and polyphenols in preventing and palliating metabolic alterations and other physiological situations governed by oxidative stress and inflammation processes to provide solid scientific evidence for the optimum design of nutritional strategies.

### 2.1. Combined Polyphenols and Fish Oils Intake for Improving Metabolic Syndrome Features

Several studies, both preclinical and clinical ones, have evaluated the effects that the combination of polyphenols and fish oils would exert on MetS issues, and they are summarized in Table 1.

#### 2.1.1. Preclinical Evidence: In Vitro and In Vivo Studies

As Table 1 shows, several studies in both cell and animal models addressed the effects of the combination between fish oil and polyphenols in restoring the redox homeostasis that is broken in MetS. Some studies found additive effects on the activation of the nuclear factor Nrf2 p45-related factor 2/Kelch-like ECH-associated protein 1 (Keap1) pathway. Particularly, the treatment with a mix of the epigallocatechin-3-gallate (EGCG) and DHA significantly activated the Nrf2/Keap1 pathway in hepatic cells [39], and the supplementation with resveratrol and fish oil showed additive effects on the Nrf2/Keap1 pathway in the myocardium and aorta of obese male Wistar rats [42], increasing the survival rate of the obese rats mainly because of the decrease in oxidative stress.

The combined intake of proanthocyanidins from grape seed extract and fish oils showed relevant additive and even synergistic effects regarding oxidative stress features in a redox proteomics study focused on the modulation of the hepatic carbonylome (which is defined as the total carbonylated proteins) in female Wistar–Kyoto rats fed an obesogenic diet [43]. In fact, only the group fed both nutraceuticals, coadministered with an obesogenic diet, showed significantly lower levels of protein carbonylation in the liver and less plasma insulin concentration. Other additive effects were detected in the coadministered group, which showed lower lipid peroxidation levels and better oxygen radical absorbance capacity (ORAC) while lowering cholesterol and tumor necrosis factor-alpha (TNFα) levels, although these effects seemed to be mainly due to the fish oil effect. Both fish oil and grape proanthocyanidins up-regulated the glutathione peroxidase (GPx) activity in plasma. Interestingly, a synergistic effect on the GPx activity was measured, since the double supplementation dramatically enhanced the activity of plasma GPx, an increment that cannot be explained by the single addition of individual effects of the nutraceuticals. Moreover, both nutraceuticals and their combination tightly regulated the liver carbonylome. Some of the carbonylated proteins identified differently responded to each nutraceutical, and curiously that behavior was strongly dependent on the background diet. The functional analysis of the pathways potentially modulated by the nutraceuticals through changing carbonylation profiles revealed enrichments in pathways related to lipid and carbohydrate metabolism, antioxidant defense, and insulin signaling, which could contribute to explain the biochemical measurements. As a result, the double supplementation was the most effective in improving the metabolic health status of the rats. Apart from the effects on the redox homeostasis in the liver, it is noteworthy that the coadministration of grape seed proanthocyanidins and an oil-rich in DHA in the diet of obese male Wistar rats activated muscle β-oxidation, increased the mitochondrial functionality and oxidative capacity, and up-regulated fatty acid uptake gene expressions, mainly because of increasing 5′-AMP-activated protein kinase (AMPK) phosphorylation, up-regulating peroxisome proliferator-activated receptor alpha (PPARα) and uncoupling protein 2 (Ucp2) [44]. This study reflected that the combination of polyphenols and fish-derived omega-3 PUFAs could also improve muscle status, avoiding the detrimental effects of obesity through altering the AMPK signaling pathway, which is one of the mechanisms proposed to be behind the MetS and its derived diseases [67]. Other authors have studied the combination of fish oil with polyphenols from apples when added to a high cholesterol diet in male Sprague–Dawley rats [45]. They found that the combination of fish oil and apple polyphenols in the diet improved serum and liver lipid profiles, but the best results regarding oxidative stress were given by the supplementation with fish oil alone, although the antioxidant outcomes of fish oil remained in the double supplemented group.

Besides their effects in restoring the broken redox homeostasis in diet-induced metabolic disorders, several studies in both cell and animal models addressed the effects of the combination between fish oil and polyphenols on inflammation, which is also a hallmark of diet-related metabolic disorders.

The mechanisms behind the anti-inflammatory effects of resveratrol plus EPA were explored in macrophages [40], human peripheral blood leukocytes (PBLs), and articular chondrocytes from knee (NHAC-kn) [41]. Synergistic anti-inflammatory effects were detected in macrophages regarding decreased NO levels, modulation of the phospho-stress activated protein kinase/Jun N-terminal kinase (P-SAPK/JNK) level, down-regulation of proinflammatory genes, such as interleukins (IL), chemokines, transcription factors; and up-regulation of several antioxidant genes [40]. In the secretome of IL-1β activated human chondrocytes, resveratrol and EPA exerted synergistic effects on CCL5/RANTES and additive effects on IL-6 or CXCL8/IL-8. Moreover, both omega-3 and resveratrol reduced the expression of catabolic genes, including metalloproteinases (MMPs), a disintegrin-like metalloproteinase with thrombospondin motifs-4 (ADAMTS-4), IL-1β, and IL-6, in chondrocytes [41].

The study of the effect of the combined intake of polyphenols and fish oil using animal models revealed other mechanisms behind the anti-inflammatory action of these bioactive compounds. The combination of grape proanthocyanidins and fish oil [46] in female Wistar–Kyoto rats fed an obesogenic or a control diet, resulted in significant changes in both total lipid and lipid mediator profiles. The high intake of fish oil caused an enrichment in omega-3 PUFAs of membranes and tissues, with the concomitant decrease in omega-6 amount. As a consequence of this replacement, rats exhibited a more favorable inflammatory and redox status, which was defined by a shift in the 12/15-lipoxygenases activities towards omega-3 PUFAs, enhanced GPx activities, and significant modulation of the cyclooxygenase (COX)-dependent synthesis of proinflammatory lipid mediators and the down-regulation of de novo synthesis of arachidonic acid (ARA) leaded by Δ5 desaturase. Polyphenols’ bioactivity was more dependent on the background diet, being more active when added to a standard diet. In this healthy framework, the coadministration of polyphenols and fish oils cooperatively downregulated Δ5D and COX activities on ARA, enhancing the antioxidant enzymes and decreasing total FFA in plasma. In the obesogenic framework, the double supplementation significantly improved the antioxidant status, but the proinflammatory outcomes are mainly derived from the fish oils effects rather than polyphenols because the supplementation with polyphenols alone of the obesogenic diet led to the activation of some proinflammatory pathways (up-regulation COX pathways toward omega-3 proinflammatory eicosanoids as PGE2 and 11-HETE and decreased the detoxification of omega-3 hydroperoxides). Interestingly, the addition of fish oils suppressed those potentially negative effects of polyphenols in the obesogenic diet. Therefore, additive effects between fish oils and polyphenols were found in the standard diet, but fish oils are mainly behind the positive effects in obesogenic one rather than polyphenols, considering lipid mediator modulation. Moreover, the double supplemented group showed increased GPx activity, as well as monounsaturated fatty acid and polyunsaturated fatty acid-containing diacylglycerols (DAG) and long-chain fatty acid-containing ceramides abundances compared to the control [47].

These lipidomic profiles were correlated with lower insulin resistance, and further research demonstrated that there was also an up-regulation of proteins involved in improving insulin signaling as well as glycolysis enzymes, enhancing fatty acid beta-oxidation and ameliorating endoplasmic reticulum stress in the liver, especially in the double supplemented obesogenic diet [48]. Biochemical and biometric parameters confirmed the conclusions given by the lipidomic and proteomic data, which showed that whereas separate supplementation with fish oil or grape proanthocyanidins might not counteract all the metabolic disturbances induced by the obesogenic diet, the nutraceutical combination could restore insulin, leptin, and triglyceride levels to normal values [49].

Several authors also used preclinical studies to test the effects of the combination of polyphenols, marine omega-3 PUFAs, and other biologically active substances. Fish oil supplemented with plant oil extracts (from *Schisandra chinensis* and *Matricaria chamomilla*), rich in tocopherols, cholecalciferol, retinol, lignans, coumarins, and dicyclo esters, [50] demonstrated synergistic effects as free radical scavengers compared to controls in mice animal models. Brown seaweed lipids extracts (rich in polyphenols, omega-3, and fucoxanthin) resulting in less lipid peroxidation in the liver of female KK-Ay mice, although the hepatic percentage of PUFAs (especially DHA and AA) increased [51]. The long-term intake of a mixture of polyphenols, b-carotene, probiotics, and salmon fat modulated the expression of relevant genes associated with chronic disorders (gonadotrope cell activation pathway and guanylate cyclase pathway, mast cell activation, gap junction regulation, melanogenesis, and apoptosis) in the liver of male Sprague–Dawley rats. These data suggested a link between the diet, reproductive system function, and aging and the potential control by diet bioactive substances [52]. Moreover, an anti-inflammatory bioactive mixture containing resveratrol, lycopene, catechin, vitamins E and C, and fish oil was effective in improving lipid and inflammatory CVD risk factors. This nutraceutical mixture strongly reduced atherosclerotic lesion development in female transgenic mice because of decreased cytokine-induced human C-reactive protein (CRP) and fibrinogen expression, plasma cholesterol, triacylglycerols (TG), serum amyloid Aβ and expression of the vascular inflammation markers and adhesion molecules as compared to the control group [53]. Focusing on cardiovascular protection, a new functional food was developed by combining the bioactive capacities of fish oil (namely salmon oil, rich in PUFAs, omega-3, omega-6, and vitamins A, E, and D3) with a herbal oil extracted from motherwort (rich in flavonoids and iridoids) to obtain a food with superior cardioprotective properties than a single product, in terms of normalization of heart rate after ischemia, increasing the left ventricular pressure and normalizing the contraction and relaxation of the left ventricle, while decreasing aspartate amino transferase and creatine kinase activity in rats, without any toxicity [54].

#### 2.1.2. Clinical Evidence: Human Trials

The combined effect between polyphenols and fish oils was also evaluated in some human trials focusing MetS disturbances based on their synergistic, additive, or complementary antioxidant and anti-inflammatory properties supported by the studies in cell and animal models.

The effects of diets rich in fish-derived omega-3 (EPA and DHA) and polyphenols in subjects at high cardiovascular risk have been studied in several human trials focused on lipoprotein metabolism and atherogenicity. Results showed that both nutraceuticals induced: a reduction in the postprandial lipid content of large very-low-density lipoprotein (VLDL) and increases intermediate-density lipoprotein (IDL) cholesterol; modification of the composition of LDL particles, which become richer in triglycerides, and of HDL, which become instead triglyceride poor [55,56] while decreasing oxidative stress (lower urinary 8-isoprostane concentrations) [56], and blood glucose (mainly in response to polyphenols), insulin secretion and postprandial glucagon-like peptide 1 levels (mainly because of marine omega-3 intake) [57]. Moreover, nutritional intervention with diets rich in fish oils and polyphenols induced relevant lipid rearrangements, especially in the phospholipids fatty acid profiles of HDL, in subjects at high cardiovascular disease risk, which could be useful as a biomarker of early lipid metabolic alterations [58].

The beneficial influence of diets enriched in proanthocyanidins from cranberry juice and fish oil on both insulin and lipoprotein metabolisms were also found in patients with diabetes and periodontal disease since this mixture of nutrients decreased glycated hemoglobin and increased HDL-C, while improving periodontal status in those patients [59]. It was also reported that the postprandial chylomicron response to acute or chronic fish oils plus polyphenols intake is different and even opposite [60], adding an extra layer in the complex effects of the nutritional intervention with these food compounds. Some other authors demonstrated that the balanced omega-6/omega-3 ratio should also be considered in this kind of nutrition strategies, at least for patients after percutaneous coronary intervention, where it seemed that a balanced omega-6/omega-3 ratio rather than high polyphenols and fish in the diet was the main responsible for lowering some inflammation markers and predictors of CV mortality (platelet to lymphocyte ratio and CRP) in blood [61]. A significant modulation of the microbiota composition of diets naturally rich in polyphenols and fish in subjects at high cardio metabolic risk was also reported [62] and correlated with changes in glucose and lipid metabolism. Previous studies in animal models have already demonstrated the influence of fish oils in the bioavailability of proanthocyanidins by changing the microbial-derived metabolites in rats fed both bioactive compounds [21]. Interestingly, some pieces of information demonstrated that statin therapy of patients suffering from CVD could be efficiently complemented with a combination of fish oils and polyphenols, which reduced LDL-C and CRP, allowing the treatment of patients with or at risk for CVD who cannot tolerate high dose statin therapy by using lower statin dose and those specific dietary compounds [63].

Several mixtures of bioactive substances and nutraceutical cocktails, all of them especially rich in polyphenols and fish oils, were also tested in human trials. A mix of phytosterols, antioxidants, probiotics, fish oil, berberine, and vegetable proteins significantly decreased body and fat mass and improved plasma lipid profiles and inflammation markers in healthy overweight subjects with MetS that were following a proprietary lifestyle modification program [64]. One similar nutraceutical cocktail (composed of polyphenols, omega-3 fatty acids, vitamin E, and selenium) were evaluated on the metabolic and physiological changes induced by sedentary behaviors along with fructose overfeeding [66], resulting in prevention of the alterations on lipid metabolism but without any effect in preventing insulin resistance. Finally, the combination of EPA, DHA, oligomeric proanthocyanidins, resveratrol, and vitamin K2, vitamin B6, vitamin B12 (Aterofisiol^®^) [66] altered the composition of the atherosclerotic plaque significantly, reducing the amounts of cholesterol and lipids and providing more protection against the adverse neurological events that could be derived from the rupture of the atherosclerotic plaque.

### 2.2. Combined Polyphenols and Fish Oils Intake in Neurodegenerative Pathologies, Cancer, and Other Health Effects

Besides MetS disorders, during the last decade, the combination of polyphenols and fish oils has been studied for the development of nutritional strategies that would result in an effective method of preventing and treating other diseases. In all the cases, these diseases, and also other physiological situations addressed, were also characterized by the disruption/change of the redox homeostasis and/or inflammatory status, and hence, they are strongly influenced by diet as well. Many of them are age-related diseases, such as brain damages, cognition alterations, and neurodegenerative diseases, which have been gained increasing interest in parallel with the extension of lifespan and the dramatic rate of aging of the worldwide population. The summary of the studies addressing these issues from the last decade is shown in Table 2.

#### 2.2.1. Preclinical and Clinical Evidence on Neurodegenerative Diseases

In order to discover the mechanistic roles behind the beneficial effects of polyphenols and fish oils on cognition alteration and neurodegenerative processes, it has been investigated the effects of dietary supplementation with resveratrol and DHA on hippocampal gene expression in adult C57BL/6 mice because the hippocampus is vital for multiple cognitive functions, including memory formation [70]. Most of the genes that were significantly altered were associated with inflammatory responses, with a decreased IL-6 and apolipoprotein E expression. So, genomic data indicate that resveratrol and DHA likely exert their beneficial effects through anti-inflammatory mechanisms.

This anti-inflammatory action was further investigated by determining the role of polyphenols and fish oils in modulating the release of proinflammatory mediators and cytokines related to cognitive failures and neurodegenerative processes. In cell models, [68] it was reported that low concentrations of a combination of fish oils (particularly EPA-rich) and phytonutrients (Lyc-O-mato, carnosic acid with or without lutein) were very efficient in inhibiting the transformation of microglia to proinflammatory M1 phenotype and may prevent cognition deficit because there were synergistic effects in the inhibition of the redox-sensitive NF-κB (nuclear factor-κ light-chain enhancer of activated B-cell) activation and increasing the anti-inflammatory IL-10 secretion. In N2a neural cell, it was also demonstrated that the combination of polyphenols (resveratrol, quercetin, apigenin), as well as omega-3 and omega-9 unsaturated fatty acids (α-linolenic acid, ALA, EPA, DHA, and oleic acid (OA)) main nutrient of the Mediterranean diet, were potent cytoprotective agents against 7-Ketocholesterol-induced neurotoxicity by decreasing ROS, mitochondrial dysfunction and cell death [69]. The benefits of other diets rich in polyphenols and polyunsaturated fatty acids, like the LMN diet, have also been shown in animal models of aging and Alzheimer’s disease, where the diet decreased the behavioral deterioration and delayed the amyloid plaques formation [71], and caused an enhanced modulatory effect on both cholinergic and catecholaminergic transmissions [72] pointing toward to a possible reducing effect on cognitive decline underlying aging and Alzheimer’s disease. Similar conclusions on the improvement of memory and cognitive performance were also reached in animal models feeding diets rich in those compounds and other bioactive nutrients [74].

The supplementation of the diet with Smartfish^®^, a bioactive mixture containing omega-3 EPA and DHA, resveratrol, vitamin D, and whey protein, increased amyloid-β phagocytosis and resolvin D1 in patients with minor cognitive impairment [75]. However, a further clinical trial with this diet in a sample from older adults (68–83 years) without any other pathology, had only a limited beneficial impact, highlighting the need for more investigations to establish any potential clinical applications of such targeted interventions with longer durations of supplementation, or in populations with defined cognitive deficits [76].

Several other studies have addressed the effect of omega-3 and polyphenols in young subjects. It has been shown that the combination of resveratrol, prebiotic fiber, and omega-3 DHA improves the post-mild traumatic brain injury function in adolescent rats [73]. In addition, supplementary feeding for 23 weeks with plant polyphenols and fish oils seemed to improve executive function, brain health, and nutritional status in vulnerable young children at risk of undernutrition living in low-income countries [77].

#### 2.2.2. Preclinical and Clinical Evidence on Cancer

The bioactivity of the combination between polyphenols and omega-3 PUFAs was also tested against some types of cancers. Both natural lipophilic polyphenols and omega-3 PUFAs are well-known suppressors of the Wnt- and NF-κB-related pathways, at least, individually, and often have been targeted in colon cancer research, because the majority of colon tumors are driven by aberrant Wnt signaling in intestinal stem cells [90]. It has been studied the less-known effects of these nutrients (fish oil and curcumin) on colonic leucine-rich repeat-containing G-protein-coupled receptor 5-positive (Lgr5 + ) stem cells in mice, which are the cells of origin of colon cancer. The authors reported that only the combination between the bioactive compounds synergistically increased targeted apoptosis in DNA damaged Lgr5+ stem cells, maximally reduced damaged Lgr5+ stem cells, drop to the level measured in saline-treated mice. Moreover, p53 signaling in Lgr5+ stem cells from mice exposed to azoxymethane (a genotoxic carcinogen) was uniquely up-regulated only following fish oil plus curcumin cotreatment [78]. This promising combination between omega-3 and polyphenols in colorectal cancer has driven new strategies to ameliorate the efficacy and reduce the toxicity of the drugs. Particularly, the encapsulation of omega-3 PUFA into solid lipid nanoparticles (SLN) having a lipid matrix containing resveratrol esterified to stearic acidenhanced their incorporation in human HT-29 CRC cells in vitro significantly, and also their growth inhibitory effects in these cancer cells because of reduced cell proliferation [91].

Other nutraceutical combinations were also evaluated for different cancers. A recent study reported that olive-derived polyphenol hydroxytyrosol combined with omega-3 fatty acids and curcumin (PureVida™) reduced inflammation and pain in patients with aromatase-induced musculoskeletal symptoms, as one of the side effects of the hormonal therapies for breast cancer [79]. Potential links between diets rich in polyphenols and fish oils, such as the Mediterranean diet, were also described for prostate cancer. The high adherence to this diet was found inversely associated with the likelihood of suffering from prostate cancer [80].

#### 2.2.3. Preclinical and Clinical Evidence on Other Pathologies and Physiological Processes

During this last decade, other physiological processes in which oxidative stress and/or inflammation are causal were also studied. The combination of fish oil and curcumin prevented skeletal muscle atrophy due to a boost of heat shock proteins and anabolic signaling in an unloaded state [81]. Functional beverages based on almonds and olive oil and enriched with α-tocopherol and DHA [82] protected against oxidative damage, although it enhanced nitrative damage in young athletes. These beverages enhanced the gene expression of antioxidant enzymes in peripheral blood mononuclear cells (PBMCs) after exercise in young athletes while enhanced a proinflammatory circulating environment in response to the exercise, but this proinflammatory outcome was less evident in the senior athletes [83]. The effects of an antioxidant/anti-inflammatory cocktail in the prevention of muscle deconditioning induced by long-term inactivity in healthy men were evaluated but did not find any significant one [84]. Therefore, it seems that redox balance mechanisms in skeletal muscle are extremely complex, and it is probable they were deficiently studied for the development of successful nutritional strategies.

Several studies have focused on some age-related eye diseases to test the effects of the combination between fish oils and polyphenols. As a result of these researches, it has been reported that omega-3 and resveratrol could prevent aged retinal pigment epithelial (RPE) cells damage and age-related macular degeneration [85], choroidal neovascularization [86], and blueberry polyphenols can prevent lipid peroxidation of DHA and ARA induced daily visible light exposure to the retina [92]. It has been proposed that they can be used for designed dermatologic diets to improve skin barrier function for the treatment of dermatitis [87].

Finally, some studies were aimed to test the effects of maternal supplementation with polyphenols and omega-3 on immune priming in the newborn [88], and maternal and neonatal microbiota, and the consequence of this modulation on the infant health outcomes [89]. These studies revealed that microbiota and immune priming in the newborn are tightly regulated by maternal diet composition and polyphenols and omega-3 particularly, affecting microbiota populations and modulating the histone acetylation pattern in placentas. Previous studies already demonstrate that a combination of polyphenols, particularly resveratrol and marine omega-3 EPA and DHA, modulated the gene expression level of histone deacetylase Sirtuin 1 in human monocytes (THP1) [93].

### 2.3. Omega-3 Lipophenols Derivatives: The Combined Properties of Omega-3 PUFAs and Polyphenols in a Single Molecule

The successful combination of the biological activities of polyphenols and omega-3 PUFAs on oxidation, inflammation, diet-related diseases, cancer, and other human pathologies have been reported in several studies, as we showed in this review. As a consequence, the interest in the so-called omega-3 lipophenol derivatives or phenolipids has grown during the last decade. It has been described a heterogeneous group of different chemical structures obtained from the chemical bonding between polyphenols and omega-3 PUFAs and some from natural sources [94,95,96]. There are also esterified phenols with fatty acids different from omega-3 PUFAs, such as caprylic acid [97], palmitoyl acid [98], or oleic acid [99], mainly investigated because of their protective effect in lipid-based food matrices from oxidation, and more recently as functional food ingredients [100]. Even if they are also very relevant and showed important properties and functions, they are out of the scope of this review, and only the last studies about omega-3 lipophenol derivatives will be considered and summarized in Table 3.

Over the last five years, esterified phenols with DHA have been studied for the treatment of retinal pathologies characterized by high levels of carbonyl and oxidative stresses, including age-related macular degeneration and Stargardt’s disease. Several lipophenols has been synthetized and tested, such as O-alkylated resorcinol derivatives (phloroglucinol or resveratrol) linked to DHA or lysophosphatidylcholine–DHA [23], isopropyl-phloroglucinol–DHA [101,102,108], quercetin conjugated to DHA [103], and very recently, a new DHA-quercetin lipophenol [104]. These investigations reported powerful antioxidant properties and high protection activities against reactive aldehyde all-trans-retinal toxicity and photo-oxidative toxicity and constitute highly promising strategies for the prevention of retinal degeneration. Antioxidant, anti-inflammatory, and anticarcinogenic properties for quercetin and quercetin glycosylated derivatives bound to omega-3 PUFAs ALA, EPA and DHA were also reported in lung cells exposure to H_2_O_2_ insult [105] or cigarette smoke toxicants [106].

Finally, DHA and phenols conjugated have also been synthesized for increasing the bioactivity of the individual compounds against breast cancer. Remarkably, DHA-acylated phloridzin (PZ-DHA), a novel polyphenol fatty acid ester derivative [109], was selectively cytotoxic to breast cancer cells in vitro and in vivo and also might prevent or inhibit the progression of triple-negative breast cancer (TNBC) [110]. DHA linked to resveratrol was evaluated as an inhibitor of gelatinolytic matrix metalloproteinase MMP-9, but its inhibitory activity was lower than the resveratrol-linoleic acid conjugated [107].

## 3. Conclusions

The health benefits of the combination of the bioactive properties of polyphenols and fish-derived omega-3 PUFAs have been largely supported by a growing amount of scientific evidence. Much more research is still needed to fully understand the biological mechanisms behind the health effects of those nutraceuticals because of the complexity of the interactions between nutrients and metabolism. Nevertheless, researchers have made huge efforts to unveil those mechanisms, and some of them have been already described. Significant cooperative antioxidant and anti-inflammatory effects have been consistently reported. The antioxidant outcomes seem to be mediated by the additive effects of both nutraceuticals on the Nrf2 signaling pathway and synergistic effects on the activity of glutathione peroxidase, which triplicated its activity in some animal models. Amplification of the plasma antioxidant capacity (more than double in regards to individual capacities) and attenuation or even antagonism of lipid peroxidation, protein carbonylation, and urinary 8-isoprostane (decreases of up to 50–100% regarding individual effects) were other antioxidant cooperative effects often reported. Regarding inflammation, the combination of the individual bioactivities showed additives effects on several inflammation biomarkers, such as TNFα, PGE2, or IL-6, which was attenuated by 50–200% in some studies. Nutraceuticals also induced lipid rearrangements, changed total lipid profiles, and altered the formation of lipid mediators towards anti-inflammatory profiles. Alterations in the metabolism of the lipoproteins and chylomicrons, formation of amyloid Aβ, and atherosclerotic plaques are also consistently reported. Improvements in insulin sensitivity mediated by changes in protein regulation and gene expression and also gut microbiota modulation were also reported. It should be noted that the bioactive properties of polyphenols are closely dependent on their chemical structure, and the proportion between EPA and DHA determines their functional activities. Among other multiple factors that can influence the results and the different conclusion extracted from several studies, this review highlight that the bioactivity of those nutraceuticals are strongly influenced by the background diet, the progression of the pathophysiological condition, the physical activity of patients and age, the presence of other bioactive substances, etc. As a consequence, some bioactive cocktails failed in reproducing the beneficial effects described in cell and animal models when they were administered in human trials. Because of the complexity of the interaction of nutraceuticals-metabolism and multiple mechanisms of action, future research should adopt multi-omic approaches and systems biology strategies to fully understand that interaction and mechanisms and identify novel and more precise biomarkers. Current research also demands more clinical trials and a careful selection of the population cohort in each case. Moreover, lipophenols, which combine the bioactive properties of both nutraceuticals in a single molecule, are a very promising strategy for the treatment and prevention of a plethora of human diseases that need to be tested, and their health effects also need to be validated both in in vivo studies and human trials.

To summarize, the combination of the bioactive properties of the polyphenols and fish oils have demonstrated important beneficial effects, especially as antioxidant and anti-inflammatory agents, because of several synergistic, additive, or complementary effects between both nutraceuticals, as is evidenced in the majority of the studies already published. However, more information is needed to understand how these nutraceuticals interact with each other in vivo and also how they actually interact with the metabolism of a certain organism, and if it is wanted to use those nutraceuticals as part of personalized nutrition.

## Figures and Tables

**Table 1:** Summary of the researches from the last ten years that have studied the effect of the combination between polyphenols and fish oils for improving Metabolic Syndrome features.

Bioactive	Dose	Model	Health Effects of the Combination	Reference
In vitro studies				
Epigallocatechin-3-gallate (EGCG) from green tea and DHA.	EGCG, DHA or EGCG + DHA (50 µM) 1 h.	FaO cells (H4-11-E-C3 rat hepatoma).	Less lipid peroxidation levels More GSH/GSSG and less catalase EGCG impairs DHA-related Nrf2 nuclear translocation and decreases HO-1 protein levels.	[39]
Resveratrol and EPA.	Resveratrol (25 mg/mL); EPA (30 mM); 19 h.	RAW 264.7 murine macrophage.	Enhanced anti-inflammatory effect Decreased NO levels; Modulating P-SAPK/JNK; Down-regulation of proinflammatory; genes (IL, chemokines, transcription factors); Up-regulation antioxidant genes.	[40]
Resveratrol and EPA.	Resveratrol(25 µmol/L); EPA (20 µmol/L).	Human peripheral blood leukocytes (PBLs); Normal human articular chondrocytes from knee (NHAC-kn).	Synergistic effects on CCL5/RANTES; Additive effects on IL-6 or CXCL8/IL-8.	[41]
In vivo studies				
Resveratrol and fish oil.	20 mg resveratrol/kg/day; 0·4 g fish oil (54% EPA, 10% DHA)/kg per day; 2 months.	Obese male Wistar rats.	Activation of the Nrf2/Keap1 pathway; Increases survival of obese rats because of less oxidative stress in the aorta and myocardium.	[42]
Grape seed proanthocyanidins extract and fish oil.	Proanthocyanidin rich grape seed extract (GSPE, 0.8 g kg^−1^ feed) EPA/DHA 1:1 (16.6 g kg^−1^ feed); 24-weeks.	Prediabetic female Wistar–Kyoto rats.	Both additive and synergistic effects on total and specific protein carbonylation in liver; Effects strongly depended on the background diet; Results correlated with improved insulin sensitivity and antioxidant status.	[43]
Grape seed proanthocyanidins extract and oil rich in DHA.	GSPE (25 mg/kg body weight); 500 mg oil-rich DHA (38.8%)/kg body weight; 21 days.	Obese male Wistar rats.	Activation of muscle β-oxidation More mitochondrial functionality and oxidative capacity; Up-regulation of AMPK phosphorylation, PPARα and Ucp2.	[44]
Apple polyphenols and fish oil.	1.5% apple polypheno l10% fish oil (27% EPA, 11% DHA); 4 weeks.	Male Sprague–Dawley rats.	Synergistic effects: lower posterior abdominal fat wall and testicle peripheral fat; Additive effects: lower cholesterol and FFA; lower adiponectin than in fish oil and more than in polyphenols; less oxidative stress than in polyphenols but more than in fish oil.	[45]
Grape seed proanthocyanidins extract and fish oil.	GSPE(0.8 g kg^−1^ feed) EPA/DHA 1:1 (16.6 g kg^−1^ feed); 24-weeks.	Prediabetic female Wistar–Kyoto rats.	Complementary effects: Lower omega-6/-3 ratio; Lower production of ARA proinflammatory lipid mediators; Up-regulation desaturases towards omega-3. Additive effects: Down-regulation Δ5D and COX activities on ARA; Enhancing the antioxidant enzymes decreasing total FFA in plasma.	[46]
Grape seed proanthocyanidins extract and fish oil.	GSPE(0.8 g kg^−1^ feed) EPA/DHA 1:1 (16.6 g kg^−1^ feed); 24 weeks.	Prediabetic female Wistar–Kyoto rats.	Synergistic effect of GPx activity; Higher amount of MUFA and PUFA-containing DAG and long-chain fatty acid-containing ceramides.	[47]
Grape seed proanthocyanidins extract and fish oil.	GSPE(0.8 g kg^−1^ feed) EPA/DHA 1:1 (16.6 g kg^−1^ feed). 24 weeks.	Prediabetic female Wistar–Kyoto rats.	Additive effects on the regulation of proteins involved in insulin signaling, glycolysis, fatty acid beta-oxidation, and endoplasmic reticulum stress.	[48]
Grape seed proanthocyanidins extract and fish oil.	GSPE(0.8 g kg^−1^ feed) EPA/DHA 1:1 (16.6 g kg^−1^ feed); 24 weeks.	Prediabetic female Wistar–Kyoto rats.	Additive effect on insulin, leptin, and triglycerides levels in prediabetic rats.	[49]
Plant oil extracts (tocopherols, cholecalciferol, retinol, lignans, coumarins and dicyclo esters) and fish oil.	Daily oral gavage of salmon oil (1365 mg/kg body weight) supplemented with *Schisandra chinensis* oil extract and *Matricaria chamomilla* oil extract at growing doses of plant extract from 1365, 2730 to 5460 mg/kg body weight; 21 days.	Male Balb/c mice.	Synergistic antioxidant effect as free radical scavengers; Better immunomodulatory activity at highest plant extract doses without any toxicity.	[50]
Brown seaweed lipids.	0.5% or 2.0% seaweed lipids; 4 weeks.	Female KK-Ay mice.	Less lipid peroxidation in the liver; Hepatic enrichment in DHA and ARA.	[51]
Anti-inflammatory dietary mixture (AIDM) (resveratrol, lycopene, catechin, vitamins E and C, and fish oil)	AIDM; 6 weeks.	Female ApoE*3Leiden transgenic mice.	Decreased CRP and fibrinogen expression. Decreased plasma cholesterol, TG, serum amyloid Aβ, vascular inflammation markers, and adhesion molecules	[52]
Biologically active substances-enriched diet (BASE-diet) (polyphenols, b-carotene, probiotics, and omega-3 and -6 PUFAs).	BASE-diet; 3 vs. 14 months	Adult male Sprague–Dawley rats.	Regulation of gonadotrope cell activation pathway and guanylate cyclase pathway, mast cell activation, gap junction regulation, melanogenesis, and apoptosis.	[53]
Functional food of salmon oil (omega-3 and omega-6 PUFAs, vitamins A, E and D3) with oil extract of motherwort (flavonoids and iridoids).	Daily intragastric administration of functional food (salmon oil:motherwort oil extract in 8:2 ratio) at the doses of 2340 and 1170 mg/kg body weight; 14 days.	Rats.	Increased left ventricular pressure after ischemia; Normalized contraction/relaxation of left ventricle; Decreased aspartate amino transferase and creatine kinase activity; Cardioprotective effect without any toxicity.	[54]
Human studies				
Polyphenols from green tea and coffee, vegetables, fruits, dark chocolates, and extra-virgin olive oil; Omega-3 PUFAs from salmon, dentex, and anchovies.	Diet naturally rich/or not in omega-3 PUFAs (4 g/day) and/or polyphenols (2.861 mg/day); 8 weeks	Humans at high metabolic risk.	Reduction of the postprandial lipid VLDL; Increases IDL; LDL richer and HDL poorer in TG.	[55]
Polyphenols from green tea and coffee, vegetables, fruits, dark chocolates, and extra-virgin olive oil; Omega-3 PUFAs from salmon, dentex, and anchovies.	Diet naturally rich/or not in omega-3 PUFAs (4 g/day) and/or polyphenols (2.861 mg/day); 8 weeks.	Humans at high metabolic risk.	Additive effects of polyphenols (less TG, large VLDL, and urinary 8-isoprostanes) and of fish oils (less postprandial chylomicron cholesterol and VLDL apolipoprotein B-48); Correlation lipoprotein changes and 8-isoprostanes.	[56]
Polyphenols from green tea and coffee, vegetables, fruits, dark chocolates, and extra-virgin olive oil; Omega-3 PUFAs from salmon, dentex, and anchovies.	Diet naturally rich/or not in omega-3 PUFAs (4 g/day) and/or polyphenols (2.861 mg/day); 8 weeks	Humans at high metabolic risk.	Additive effects of polyphenols (less plasma glucose and increased early insulin secretion) and of fish oils (reduced beta-cell function and GLP-1).	[57]
Polyphenols from green tea and coffee, vegetables, fruits, dark chocolates, and extra-virgin olive oil; Omega-3 PUFAs from salmon, dentex, and anchovies.	Diet naturally rich/or not in omega-3 PUFAs (4 g/day) and/or polyphenols (2.861 mg/day); 8 weeks.	Humans at high metabolic risk.	Lipid rearrangements (in phospholipids fatty acid profiles of HDL).	[58]
Cranberry polyphenols; EPA and DHA.	200 mL of the cranberry; 1 g omega-3 fatty acid capsule, 180 mg EPA and 120 mg DHA, twice daily; 8 weeks.	Humans with diabetes and periodontal disease.	Decreased glycated hemoglobin; Increased HDL-C; Improve periodontal status.	[59]
Polyphenols from green tea and coffee, vegetables, fruits, dark chocolates, and extra-virgin olive oil; Omega-3 PUFAs from salmon, dentex, and anchovies.	Diet naturally rich/or not in omega-3 PUFAs (4 g/day) and/or polyphenols (2.861 mg/day); Blood samples taken before and up to 6 h after the test meal.	Humans at high metabolic risk.	Change in levels of chylomicron cholesterol and triglycerides due to omega-3; Response to nutraceuticals depends on acute or chronic supplementation.	[60]
Diet rich in polyphenols and omega-3; PUFAs.	Retrospective study; June 2017 to December 2018; Łódź, Poland.	Middle-age patients after percutaneous coronary intervention.	PLR and NLR depending on the omega-6/omega-3 ratio.	[61]
Polyphenols from green tea and coffee, vegetables, fruits, dark chocolates, and extra-virgin olive oil; Omega-3 PUFAs from salmon, dentex, and anchovies.	Diet naturally rich/or not in omega-3 PUFAs (4 g/day) and/or polyphenols (2.861 mg/day); 8 weeks.	Human at high metabolic risk.	Change in gut microbiota associated with changes in glucose/lipid metabolism.	[62]
Fish oil; Chocolate containing plant sterols and green tea.	Fish oil (1.7 g EPA + DHA/day); Chocolate containing plant sterols (2.2 g/day); Green tea (two sachets/day); 6 weeks.	Patients suffering from type 2 diabetes.	Both nutraceuticals combined with statin therapy significantly reduced LDL-C and CRP.	[63]
Mix of phytosterols, antioxidants, probiotics, fish oil, berberine, and vegetable proteins (PROG) + proprietary lifestyle.	PROG plan daily; 13 weeks.	Healthy overweight people with cardiometabolic syndrome.	Less body and fat mass; Improved plasma lipid profiles and inflammation markers.	[64]
Nutraceutical cocktail (polyphenols, omega-3 fatty acids, vitamin E, and selenium).	Nutraceutical cocktail daily; 10–20 days.	People with sedentary behaviors and fructose overfeeding.	Less alterations on lipid metabolism; No effect in preventing insulin resistance.	[65]
Aterofisiol^®^ (EPA, DHA, oligomeric proanthocyanidinsand resveratrol, vitamins K2, B6, and B12).	Aterofisiol^®^; 1 tablet every 24 h starting 30 days before the surgery and stopping 5 days before it.	Patients with carotid stenosis who underwent endarterectomy.	Alteration of atherosclerotic plaque composition; More prevention from neurological events associated.	[66]

**Table 2:** Summary of the researches from the last ten years that have studied the effect of the combination between polyphenols and fish oils for improving neurodegenerative pathologies, cancer, and other health effects.

Bioactive	Dose	Model	Health Effects of the Combination	Reference
**Neurodegenerative Diseases**				
In vitro studies				
EPA, Lyc-O-mato, carnosic acid, and lutein.	0.125 µM EPA, 0.1 µM Lyc-O-mato, 0.2 µM carnosic acid and 0.2 µM lutein.	BV-2 immortalized murine microglial cell line.	Synergistic inhibition of the production of proinflammatory mediators: Inhibition redox-sensitive NF-κB activation; Inhibition of superoxide production; Upregulation COX-2 and iNOS; More release of PGE2 and NO; Attenuation IL-6 and CD40.	[68]
Polyphenols (resveratrol, quercetin, and apigenin), omega-3 and omega-9 fatty acids (α-ALA, EPA, DHA, and OA) and α-tocopherol.	Polyphenols: 1.5 to 6.25 µM; Fatty acids: 6.25 to 50 µM. α-Tocopherol: 400 µM.	N2a Neuronal cells.	Cytoprotective against 7-Ketocholesterol-induced neurotoxicity.	[69]
In vivo studies				
Resveratrol and DHA.	50 mg/kg/day of each supplement (alone and combined); 6 weeks.	Adult C57Bl/6 mice.	Modulation of steroid hormone biosynthesis, JAK-STAT signaling pathway, ribosome, graft-versus-host disease pathways in the hippocampus; Decreased IL-6 and Apolipoprotien E (ApoE) expression.	[70]
LMN diet rich in polyphenols and PUFAs.	LMN diet; 5 months.	Tg2576 male and female mice as a model of AD.	Delays the Aβ plaque formation and decreases Aβ_1–40_ and Aβ_1–42_ plasma levels in adult mice.	[71]
LMN diet rich in polyphenols and PUFAs.	LMN diet; 10, 20, 30, or 40 days.	129S1/SvImJ adult male mice.	Enhancement of cholinergic and catecholaminergic transmissions; Nrf2 activation and increased protein levels of SOD-1 and GPx.	[72]
Resveratrol, prebiotic fiber, and DHA.	Resveratrol 50 mg/L drinking water; DHA and prebiotics in powdered food (100 g of prebiotic, 300 g of DHA, and 600 g of standard diet per 1 kg of food); Administration from post-natal day 21 to 43.	Adolescent male and female Sprague–Dawley rats suffering from mild traumatic brain injury.	Modify premorbid characteristics Prevented injury-related deficits in longer-term behavior measures, medial prefrontal cortex spine density, and levels of *Aqp4, Gfap, Igf1, Nfl,* and *Sirt1* expression in the prefrontal cortex.	[73]
Multivitamins, zinc, polyphenols, omega-3 fatty acids, and probiotics.	Bioactive mixture for 2 two weeks; 48 days.	Crickets.	A combination of multivitamins, zinc, and omega-3 fatty acids was the most effective for improving memory and cognitive performance.	[74]
Human studies				
Smartfish^®^ (omega-3 EPA and DHA, and resveratrol, vitamin D, and whey protein).	200 mL/day Smartfish^®^ drink containing 1000 mg DHA, 1000 mg EPA, pomegranate and chokeberry, 10 mg vitamin D3 and resveratrol, whey protein, fiber, and fruit juice; 4–17 months.	Patients with minor cognitive impairment (MCI), with pre-MCI, or with Alzheimer disease (AD).	Increase amyloid-β phagocytosis and resolvin D1 in patients with MCI.	[75]
Smartfish^®^ (omega-3 EPA and DHA, and resveratrol, vitamin D, and whey protein).	200 mL/day Smartfish^®^ drink containing 1500 mg DHA and 1500 mg EPA, 10 μg vitamin D3, 150 mg resveratrol, and 8 g whey protein isolate; 6 months.	Older adults (68–83 years) without any specific pathology.	Limited beneficial effects improving cognitive function.	[76]
NEWSUP (high in plant polyphenols and omega-3 fatty acids, high fortification of micronutrients, and high protein content).	NEWSUP; 23 weeks.	Children aged 15 months to 7 years; primary population: children younger than 4.	Increased working memory, hemoglobin concentration among children with anemia, decreased body mass index z score gainm, and increased lean tissue accretion with less fat; Increased index of cerebral blood flow (CBFi).	[77]
**Cancer**				
In vivo studies				
Curcumin and fish oil.	1% (*w*/*w*) curcumin; 4% (*w*/*w*) menhaden fish oil; 3 weeks nutraceutical supplementation + genotoxic carcinogen injections + 17 weeks.	Lgr5-EGFP-IRES^creERT2^ knock-in mice.	Only fish oils + curcumin reduced nuclear β-catenin in aberrant crypt foci and synergistically increased targeted apoptosis in DNA damaged Lgr5+ stem cells; Only fish oils + curcumin up-regulated p53 signaling in Lgr5+ stem cells from mice exposed to a carcinogen.	[78]
Human studies				
PureVida™ (EPA/DHA/hydroxytyrosol/curcumin).	3 capsules of PureVida™/day; Each capsule: 460 mg of fish oil (EPA and DHA), 125 mg of Hytolive^®^ powder (12.5 mg of natural hydroxytyrosol), and 50 mg extract of curcumin (47.5 mg curcuminoids); 1 month.	Post-menopausal breast cancer patients.	Decrease in CRP; Reduction of pain from aromatase inhibitors of hormonal therapies.	[79]
Mediterranean diet.	Mediterranean-type dietary pattern; Population-based case–control study; January 2015 to December 2016; Catania, Italy.	Prostate cancer (PCa) cases and controls.	High adherence to diet inversely associated with the likelihood of prostate cancer: PCa cases consume a lower amount of vegetables, legumes, and fish.	[80]
**Exercise and physical activity**				
In vivo studies				
Fish oil and curcumin.	5% fish oil (EPA: 13.2%; DHA: 8.6%; DPA: 4.9%), 1% curcumin in diet; 10 days supplement + 7 day hindlimb unloading.	C57BI/6 mice.	Decreased loss of muscle cross-sectional area; An enhanced abundance of HSP70 and anabolic signaling (Akt phosphorylation, p70S6K phosphorylation) while reducing Nox2.	[81]
Human studies				
Beverages based on almonds and olive oil and enriched with α-tocopherol and DHA.	1 L daily supplementation of almond and olive oil and α-tocopherol based beverage enriched with a DHA functional beverage five days a week; 5 weeks.	Young/senior male athletes.	Increased PUFAs and reduced SFAs in plasma; Increased DHA in erythrocyte; Increased blood cell polyphenol concentration in senior athletes; Protects against oxidative damage but enhances nitrative damage in young athletes; Gene expression of antioxidant enzymes in peripheral blood mononuclear cells after exercise in young athletes (GPx, CAT, and Cu–Zn SOD).	[82]
Beverages based on almonds and olive oil and enriched with α-tocopherol and DHA.	1 L daily supplementation of almond and olive oil and α-tocopherol based beverage enriched with a DHA functional beverage five days a week; 5 weeks.	Young/senior male athletes.	Increased TNFα levels depending on age and exercise;Attenuated the increase in plasma NEFAs, sICAM3 and sL-Selectin induced by exercise; Exercise increased PGE2 plasma levels in supplemented young athletes; Exercise increased NFkβ-activated levels in PBMCs mainly in supplemented young athletes.	[83]
Antioxidant/anti-inflammatory cocktail (polyphenols, vitamin E, selenium, and omega-3).	Daily antioxidant/anti-inflammatory cocktail (741 mg of polyphenols, 138 mg of vitamin E, 80 μg of selenium, and 2.1 g of omega-3); 60 days of hypoactivity.	Healthy, active male subjects.	Ineffectiveness regarding oxidative muscle damage, mitochondrial content, and protein balance and a disturbance of essential signaling pathways (protein balance and mitochondriogenesis) during the remobilization period.	[84]
**Age-related eye disease**				
In vitro studies				
Resvega (30 mg of trans resveratrol and 665 mg of omega-3 EPA and DHA, among other nutrients).	288 ng of Resvega (30 mg of trans resveratrol and 665 mg of omega-3, among other nutrients); 48 h.	ARPE-19 cells.	Induced autophagy by increased autolysosome formation and autophagy flux; Change p62 and LC3 protein levels; Cytoprotection under proteasome inhibition	[85]
In vivo studies				
Resvega (30 mg of trans resveratrol and 665 mg of omega-3, among other nutrients).	100 µL of Resvega once a day; 38 days.	C57BL6/J mice.	Less vascular endothelial growth factor (VEGF) protein expression levels and less MMP-9 activity; Mitigate choroidal neovascularization and retinal disease.	[86]
**Others**				
Dermatologic food (EPA + DHA + polyphenols).	Dermatologic food; 8 weeks.	Adult atopic dog.	Reductions in clinical scores of atopic dermatitis.	[87]
Olive oil polyphenols and fish oil.	Prospective birth cohort Assessment of Lifestyle and Allergic Disease During INfancy (ALADDIN) Families recruited: September 2004–November 2007; Stockholm area, Sweden.	Placentas.	Altered histone acetylation in placentas.	[88]
Omega-3 fatty acids, and polyphenols, fiber.	Mother–neonate pairs from the prospective and observational MAMI birth cohort. Recruited: 2015–2017 Spanish–Mediterranean area; 18 months.	Gut microbiota from mother–neonate pairs.	Higher abundance of the *Ruminococcus* species in maternal gut microbiota; Higher relative abundance of *Faecalibacterium prausnitzii* considered as a biomarker of colonic health, associated with anti-inflammatory properties; Modulation of neonatal microbiota.	[89]

**Table 3:** Summary of the most recent researches that have studied the potential of lipophenols for their use in the prevention and treatment of several human diseases and pathologies.

Lipophenol	Dose	Model	Health Effects of the Combination	Reference
In vitro studies				
Isopropyl-phloroglucinol (IP)-DHA; IP–D_2_-DHA; IP–D_4_-DHA.	0–80 µM for 1 h.	ARPE-19 cells.	Reduced radical lipid peroxidation status on cells under oxidative conditions as a model of age-related macular degeneration and Stargardt’s disease.	[101]
IP-DHA.	0–80 µM for 1 h.	Primary rat RPE, mouse neural retina and human ARPE-19 cells.	Both polyphenol and PUFAs are needed for anti-carbonyl and anti-oxidative capacities; Protection against a lethal dose of all-trans-retinal; Long term protection effects.	[102]
Quercetin conjugated to DHA (Q-3-DHA).	0–80 µM for 1 h.	ARPE-19 cells.	Less toxicity that quercetin alone and better anti-carbonyl capacity.	[103]
Q-3-DHA-7OiP (quercetin-isopropyl DHA).	0–80 µM for 1 h.	ARPE-19 cells.	Highly capacity against carbonyl and oxidative stresses.	[104]
Quercetin-3-O-glucoside (Q3G)-EPA and -DHA.	1 mM for 48 h at 37 °C.	Normal diploid human fetal lung fibroblast cell line (WI-38); Fresh human normal primary hepatocytes (h-NHEPS™).	Greater cell viability upon H_2_O_2_ exposure in lung and liver; Lower production of lipid hydroperoxides under induced oxidative stress.	[105]
Quercetin-3-O-glucoside (Q3G)-EPA and -DHA.	1 mM for 48 h at 37 °C.	Normal diploid human fetal lung fibroblast cell line (WI-38).	Protection against nicotine- and Cr(VI)-induced cell death and membrane lipid peroxidation; A less inflammatory response (lesser COX-2 and PGE2).	[106]
DHA linked to resveratrol (RES-DHA).	10, 20, 40, or 80 μM for 72 h.	THP-1 monocytes.	Capacity for inhibiting MMP-9.	[107]
In vivo studies				
IP-DHA.	An intravenous injection at doses from 5 to 30 mg/kg body weight; An orally gavaged administration at doses from 40 to 150 mg/kg body weight.	Albino Abca4−/− mice.	A dose-dependently decreased light-induced photoreceptor degeneration and preserved visual sensitivity by reducing carbonyl stress in the retina;Long term protection effects.	[108]
Acylated phloridzin-DHA (PZ-DHA).	In vitro: 10, 50, 100 µM for 24 h at 37 °C; In vivo: 5 intra-tumoral injections of PZ-DHA: 0.75 mg/kg; 15-days.	Mammary carcinoma (MDA-MB-231, MDA-MB-468, 4T1, MCF-7 and T-47D) cells; Female non-obese diabetic severe combined immunodeficient (NOD-SCID) mice.	Selectively cytotoxic to breast cancer cells in vitro and in vivo.	[109]
Acylated phloridzin-DHA (PZ-DHA).	In vitro: 10 µM for 24 h at 37 °C; In vivo: Intraperitoneal injection of PZ-DHA (100 mg/kg body weight) every second day for 9 days; 17-days.	Mammary carcinoma (MDA-MB-231, MDA-MB-468, 4T1, MCF-7 and T-47D) cells; BALB/c and NOD-SCID female mice.	Potential prevention or inhibition of triple-negative breast cancer (TNBC).	[110]
